# Potential Diagnostic Value of the Differential Expression of Histone H3 Variants between Low- and High-Grade Gliomas

**DOI:** 10.3390/cancers13215261

**Published:** 2021-10-20

**Authors:** Irati Hervás-Corpión, Andrea Gallardo-Orihuela, Inmaculada Catalina-Fernández, Irene Iglesias-Lozano, Olga Soto-Torres, Noelia Geribaldi-Doldán, Samuel Domínguez-García, Nuria Luna-García, Raquel Romero-García, Francisco Mora-López, Marianela Iriarte-Gahete, Jorge C. Morales, Antonio Campos-Caro, Carmen Castro, José L. Gil-Salú, Luis M. Valor

**Affiliations:** 1 Instituto de Investigación e Innovación Biomédica de Cádiz (INiBICA), 11009 Cádiz, Spain; ihervas91@gmail.com (I.H.-C.); agandrea08@gmail.com (A.G.-O.); mycafe@telefonica.net (I.C.-F.); ilirene27@gmail.com (I.I.-L.); omst2904@gmail.com (O.S.-T.); noelia.geribaldi@uca.es (N.G.-D.); samuel.dominguez@uca.es (S.D.-G.); lunanuria1981@hotmail.es (N.L.-G.); raquel.romero.garcia@juntadeandalucia.es (R.R.-G.); franciscor.mora.sspa@juntadeandalucia.es (F.M.-L.); iriarte.marianela@gmail.com (M.I.-G.); jorgcmorales@hotmail.com (J.C.M.); antonio.campos@uca.es (A.C.-C.); carmen.castro@uca.es (C.C.); jlgilsalu@hotmail.com (J.L.G.-S.); 2 Unidad de Investigación, Hospital Universitario Puerta del Mar, Av. Ana de Viya 21, 11009 Cádiz, Spain; 3 Unidad de Gestión Clínica de Anatomía Patológica, Hospital Universitario Puerta del Mar, Av. Ana de Viya 21, 11009 Cádiz, Spain; 4 Unidad de Gestión Clínica de Neurocirugía, Hospital Universitario Puerta del Mar, Av. Ana de Viya 21, 11009 Cádiz, Spain; 5 Área de Fisiología, Facultad de Medicina, Universidad de Cádiz, Plaza Fragela, 11003 Cádiz, Spain; 6 Departamento de Anatomía y Embriología Humanas, Facultad de Medicina, Universidad de Cádiz, Plaza Fragela, 11003 Cádiz, Spain; 7 Servicio de Inmunología, Hospital Universitario Puerta del Mar, Av. Ana de Viya 21, 11009 Cádiz, Spain; 8 Área de Genética, Departamento de Biomedicina, Biotecnología y Salud Pública, Facultad de Ciencias del Mar y Ambientales, Universidad de Cádiz, 11510 Cádiz, Spain; 9 Instituto de Investigación Sanitaria y Biomédica de Alicante (ISABIAL), 03010 Alicante, Spain; 10 Laboratorio de Apoyo a la Investigación, Hospital General Universitario de Alicante, Av. Pintor Baeza 12, 03010 Alicante, Spain

**Keywords:** glioblastoma, astrocytoma, oligodendroglioma, H3.1/H3.2, H3.3, *H3F3B*, *HIST1H3F*, *HIST1H3G*, *HIST1H3J*, diagnosis, RNA-seq

## Abstract

**Simple Summary:**

In the search of the key factors that differentiate the aggressive glioblastomas from lower-grade gliomas, we determined that the variants of the structural protein of the nucleosome histone H3 show different degrees of expression. In general, high expression of H3.1/H3.2 was associated with clinical features of glioblastomas whereas high expression of H3.3 was linked to molecular alterations found in low-grade gliomas. In fact, those glioblastomas showing low expression levels of H3.1/H3.2 are highly similar to low-grade gliomas, suggesting an association with glioma aggressiveness that deserves further investigation in large cohorts.

**Abstract:**

Glioblastoma (GB) is the most aggressive form of glioma and is characterized by poor prognosis and high recurrence despite intensive clinical interventions. To retrieve the key factors underlying the high malignancy of GB with potential diagnosis utility, we combined the analysis of The Cancer Gene Atlas and the REMBRANDT datasets plus a molecular examination of our own collection of surgical tumor resections. We determined a net reduction in the levels of the non-canonical histone H3 variant H3.3 in GB compared to lower-grade astrocytomas and oligodendrogliomas with a concomitant increase in the levels of the canonical histone H3 variants H3.1/H3.2. This increase can be potentially useful in the clinical diagnosis of high-grade gliomas, as evidenced by an immunohistochemistry screening of our cohort and can be at least partially explained by the induction of multiple histone genes encoding these canonical forms. Moreover, GBs showing low bulk levels of the H3.1/H3.2 proteins were more transcriptionally similar to low-grade gliomas than GBs showing high levels of H3.1/H3.2. In conclusion, this study identifies an imbalanced ratio between the H3 variants associated with glioma malignancy and molecular patterns relevant to the biology of gliomas, and proposes the examination of the H3.3 and H3.1/H3.2 levels to further refine diagnosis of low- and high-grade gliomas in future studies.

## 1. Introduction

Gliomas constitute ~70% of primary brain cancers, most of which are aggressive glioblastomas (GBs) [1]. Clinical management of GBs is challenging due to their cellular and molecular complexity, which explains their high recurrence and poor survival (with the median overall survival of ~15 months after intensive therapies). Both diagnosis and prognosis of GB based entirely on the morphological classification are insufficient [2,3] and further implementation of appropriate molecular criteria is required as predictors of patient outcome during the progression and treatment of gliomas [4,5,6,7,8]. Noticeably, epigenetic dysregulation is thought to be important for tumorigenesis and response to treatment in gliomas and can provide important tools of clinical interest, as demonstrated in the following examples. Promoter hypermethylation of the O-6-methylguanine-DNA methyltransferase (*MGMT*) gene can predict good outcomes using the first-line chemotherapeutic agent temozolomide (TMZ) [9,10]. Mutations in arginine 132 of the tricarboxylic acid cycle component isocitrate dehydrogenase IDH1 which are associated with longer survival induce the overproduction of the 2-hydroxybutyrate metabolite, leading to the inhibition of α-ketoglutarate-dependent epigenetic modulation by Jumonji-C histone demethylases and TET hydroxymethylases [11,12]. The loss of the ATPase-helicase chromatin remodelling factor ATRX (α-thalassemia/mental retardation syndrome X-linked) disrupts the interaction of multiple epigenetic modulators (including methyl-CpG-binding MECP2, H3K27 methyltransferase EZH2 or histone variants macroH2A and H3.3) in the euchromatin–heterochromatin transition of silent genomic regions [13]; therefore, it is not surprising that the presence or absence of ATRX enables the discrimination of different survival rates within low-grade gliomas [14]. Despite the implementation of these and other markers in the clinical management of gliomas, there are still conflicts regarding the classification of these tumors that justify further biomarker screenings [15].

The nucleosome is the structural unit of chromatin and is composed of 147 bp of DNA wrapped around an octamer of histones (H2A, H2B, H3 and H4). There are three main non-centromeric histone H3 variants: the so-called canonical variants H3.1 and H3.2, which only differ from each other in a single amino acid, and the replication-independent histone variant H3.3, which differs in four/five amino acids from the previous versions. Initially reported to be associated with active transcription, H3.3 has a dual role in the organization of the genome depending on its protein interactors: ATRX/death domain-associated protein DAXX in silent regions, such as telomeres and pericentric heterochromatin, and histone cell cycle regulator (HIRA) complexes in active regions [16]. H3.3 is encoded by two genes in separate chromosomes: *H3-3A* (aka *H3F3A*) and *H3-3B* (aka *H3F3B*). At least one third of children with GB are carriers of mutations in the *H3-3A* gene, affecting either lysine 27 or glycine 34 [17]; in adults, the former mutation is confined to extremely rare cases of diffuse midline gliomas, including the diffuse intrinsic pontine gliomas (DIPG) and cerebellar GBs, with some cases bearing the K27 mutation in H3.1 (specifically in the *H3C2*/*HIST1H3B* gene) [18,19,20,21]. Although histone H3 genes are not consistently mutated in adult supratentorial GBs, Gallo et al. reported that glioma stem cells (GSCs), which are largely responsible for GB recurrence [22], exhibited a reduction in the expression of *H3-3B* mRNA in culture as a plausible mechanism explaining cellular self-renewal and tumor perpetuation [23]. In this study, we explored in detail the changes in the histone H3 variants in surgical resections of adult supratentorial gliomas as a means of improving their diagnosis, extending the analysis to the canonical variants that have not yet been extensively investigated in brain cancer.

## 2. Materials and Methods

### 2.1. Patients

The SSPA Biobank of Hospital Universitario Puerta del Mar (HUPM, Cádiz, Spain) coordinated the collection, processing, management and assignment of surgical glioma resections according to the standard procedures established for this purpose (see Table S1 for a general description of the human samples and subjects). After surgery, tumors were transferred for diagnosis according to the histological criteria in astrocytomas, oligodendrogliomas (either diffuse, grade II, or anaplastic, grade III), and GBs (grade IV) and according to the biochemical and molecular criteria (see Section 2.2 and Supplementary Materials for the molecular diagnosis); the term LGG (lower-grade glioma) was applied to grades II and III for comparison with GB. Surpluses of diagnosis were maintained in a Tissue-Tek O.C.T. compound (Leica Biosystems, Nussloch, Germany) until thawing in cold 0.1 M PBS for immediate mechanical disruption and homogenization for subsequent procedures.

### 2.2. Immunodetection Assays

The following antibodies were used: H3.1/H3.2 (1:100–1:500, ABE154, EMD Millipore, Darmstadt, Germany; 1:100–1:500, AB_2793710, Active Motif, Waterloo, Belgium), H3.3 (1:250–1:1000, 09-838; 1:100–1:500, CS207327, EMD Millipore), total H3 (1:8000, ab1791, Abcam, Cambridge, United Kingdom), laminA/C (1:500, 4777, Cell Signaling Technology, Leiden, The Netherlands), β-actin (1:10000, A5441, Sigma-Aldrich, Darmstadt, Germany), IDH1 (1:1000, 3997, Cell Signaling Technology, Leiden, The Netherlands), IDH1-R132H (1:500, MABC171, EMD Millipore; manufacturer’s dilution, MAD-000475QD, Master Diagnóstica, Granada, Spain), Ki67 (manufacturer’s dilution, MAD-000310QD, Master Diagnóstica, Granada, Spain), HP1-α (1:100, ab77256, Abcam, Cambridge, United Kingdom), HRP-conjugated secondary antibodies (1:7500, A0545 and A4416, Sigma-Aldrich, Darmstadt, Germany) and Alexa Fluor cross-adsorbed secondary antibodies (1:1000, A32744, A32766, A32754 and A32790, Invitrogen, Thermo Fisher, Madrid, Spain).

Glioma samples were homogenized in the RIPA buffer (10 mM Tris HCl, pH 8.0, 1 mM EDTA, 0.5 mM EGTA, 1% Triton X-100, 0.1% sodium deoxycholate, 0.1% SDS, 140 mM NaCl) supplemented with cOmplete EDTA-free protease inhibitor cocktail tablets (Roche, Sigma-Aldrich, Darmstadt, Germany). Protein concentration was quantified using the BCA Protein Assay following the manufacturer’s recommendations (Pierce, Thermo Fisher, Madrid, Spain). Thirty micrograms of protein per sample were loaded for Western blotting analysis according to the procedures described elsewhere [24]. Proteins were visualized using the ChemiDoc system (Bio-Rad, Madrid, Spain) and quantified by densitometry analysis using the ImageJ software (v1.53f). For signal normalization between the blots, we loaded the same external control in all the gels consisting of protein extracts from murine cerebellum as this brain area expressed detectable levels of the histone H3 variants and provided sufficient material for all assays from a single source of protein extracts. Thus, signal intensities for each sample were first normalized by the signal intensity of the corresponding external control of the same blot. Subsequently, Western blotting results for each variant were further normalized with the values of total histone H3, assuming that the distinct histone H3 variants were fractions of the total protein that could be estimated independently on potential variations of total histone H3 across gliomas.

For automated immunohistochemistry, formalin-fixed paraffin-embedded gliomas were cut into 4 µm-thick sections, adhered to Superfrost Plus slides (Thermo Fisher, Madrid, Spain) and stained using an automated staining system (BenchMark ULTRA system, Roche Tissue Diagnostics, Barcelona, Spain) according to the standard automated protocols. Binding of antibodies was detected with an UltraView Universal DAB Detection Kit (Roche Tissue Diagnostics, Barcelona, Spain), including incubation with hematoxylin and a bluing reagent, and photographed under a DM750 microscope (Leica Microsystems, Wetzlar, Germany).

For fluorescence immunohistochemistry, the remaining part of the surgical resection was fixed into 4% paraformaldehyde (2 weeks at 4 °C), cryoprotected in 30% sucrose (*w*/*v*) in 0.1 M PBS, pH 7.4 (48 h), and sliced in 20 µm-thick sections. Antigen retrieval was performed by treating the free-floating sections with a solution containing 50% (*v*/*v*) formamide and sodium citrate at 65 °C for 2 h followed by a 30 min incubation in 2N HCl at 37 °C. After that, the sections were incubated for 1 h in a blocking solution containing 2.5% (*w*/*v*) bovine serum albumin, 0.25% sodium azide and 0.1% (*v*/*v*) of Triton X-100 in PBS, followed by incubations of the corresponding primary and secondary antibodies. The nuclei were counterstained for 10 min with 0.1 mg/L of DAPI. Positive cells were detected using a confocal microscope (OLYMPUS FV1000, Barcelona, Spain).

### 2.3. RNA Extraction, RT-qPCR and RNA-seq

Total RNA from glioma tissues was extracted using a TRI Reagent (Sigma-Aldrich, Darmstadt, Germany), and the quality was determined using a Qubit RNA IQ Assay Kit in a Qubit 4 Fluorometer (Thermo Fisher, Madrid, Spain) according to the manufacturers’ instructions. Only the RNA samples with integrity values >6 were used for subsequent DNase treatment with a TURBO DNA-free Kit (Thermo Fisher, Madrid, Spain) and retrotranscription into cDNA with a RevertAid First-Strand cDNA Synthesis Kit (Fermentas, Thermo Fisher, Madrid, Spain). Quantitative PCR was performed in a Rotor-Gene 6000 Detection System (Corbett, Hilden, Germany) using PyroTaq EvaGreen qPCR Mix Plus (Cmb-Bioline, Madrid, Spain). The PCR cycling conditions were as follows: 95 °C for 15 min and 40 cycles of 95 °C for 15 s, 60 °C for 20 s, 72 °C for 20 s. Each independent sample was normalized using the *TBP* and 18S rRNA levels, and the relative quantitative values were calculated according to the 2^−ΔΔCT^ method. The sequences of all the primer pairs are provided in Table S2.

Selected DNase-treated RNA samples were sent to an external sequencing service (Unidad de Genómica, Cabimer, Sevilla, Spain). The starting amount of total RNA was 100 ng following Illumina’s recommendations for preparing the sequencing library using Illumina Stranded Total RNA Prep with Ribo-Zero Plus. Deep sequencing was performed using NovaSeq (Illumina, San Diego, CA, USA) in a configuration of paired-end and 50 bp-long reads.

### 2.4. Bioinformatics and Statistics

De novo RNA-seq fastq files were analyzed in the DRAGEN RNA Pipeline v3.7.5. (Illumina): first, the reads were aligned into the human genome (GRCh38/hg38 build) using Salmon [25], followed by differential expression analysis between selected samples using DeSeq2 [26]. The reads were visualized using IGV (Broad Institute, http://software.broadinstitute.org/software/igv/, accessed on 10 September 2021). Principal component analysis (PCA) and Volcano plotting were performed with the rgl (http://cran.r-project.org/package=rgl, accessed on 10 September 2021) and EnhancedVolcano packages (https://github.com/kevinblighe/EnhancedVolcano, accessed on 10 September 2021), respectively (R version 4.0.5).

As the supporting cohort, we used the genetics and transcriptomics information from The Cancer Gene Atlas (TCGA) consortium as deposited on the Genomic Data Commons (GDC) website (https://portalgdc.cancer.gov, accessed on 10 September 2021) and from the REMBRANDT cohort as deposited in GeneBank (accession number GSE108474). Differential expression between the GB and LGG datasets was retrieved: (i) using the pipeline described previously for the TCGAbiolinks software [27] in TCGA datasets (*n* = 166 for grade IV gliomas and *n* = 528 for gliomas of grades II and III); (ii) using the Bioconductor affy [28] and limma [29] packages in the REMBRANDT datasets (*n* = 218 for grade IV gliomas and *n* = 216 for gliomas of grades II and III) (R version 3.5.3).

Mann–Whitney U tests between two conditions (grade II or LGG vs. grade IV gliomas in the Western blotting and qPCR assays), Kendall’s and Pearson’s product moment correlations were performed using the stats package in the R environment. Survival analysis was performed using the survminer and survival packages to calculate both the Kaplan–Meier curves and the associated logrank *p*-values. For the multivariate analysis, we conducted PCA after imputing the missing values (i.e., absent values in the original diagnosis) using FactoMineR and missMDA (http://factominer.free.fr/ (accessed on 10 September 2021)), respectively (R version 4.0.5).

### 2.5. Supplementary Material and Methods

See the Supplementary Information for the full description of the additional molecular diagnosis techniques (*MGMT* promoter, 1p/19q codeletion and *EGFR* expression), generation of plasmids and transient transfection assays.

## 3. Results

### 3.1. Glioblastomas Showed the Lowest Levels of Histone H3.3 and the Highest Levels of Histone H3.1/H3.2 within Gliomas

**Figure 1:**
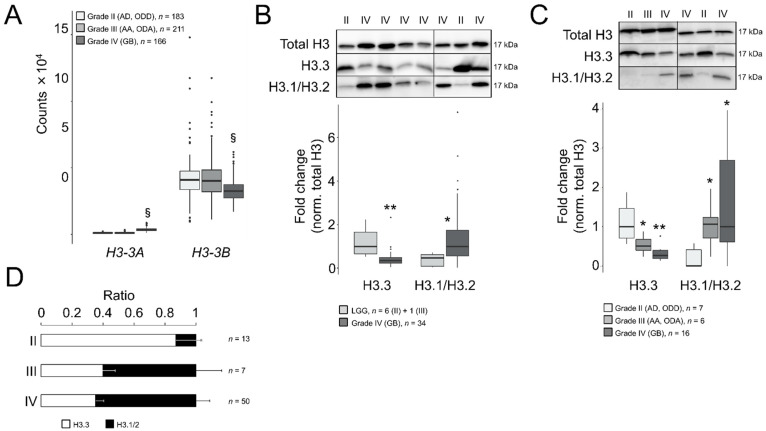
Differential ratios of H3.3 and the canonical H3 variants across gliomas of different grades. (**A**) Transcript levels of *H3-3A* and *H3-3B* in gliomas of TCGA database according to the RNA-seq analysis. Note: §, adjusted *p* < 0.005; negative binomial test. AD, diffuse astrocytoma; ODD, diffuse oligodendroglioma; AA, anaplastic astrocytoma; ODA, anaplastic oligodendroglioma; GB, glioblastoma. (**B**) Western blotting assays in surgical tumor resections of the first cohort: upper panel, representative crops of blots; lower panel, quantification. Only one sample was of grade III (OD) and was indistinguishable from grade II samples, therefore we pooled the data (LGG, lower-grade gliomas). Total H3, antibody against a common sequence epitope in histone H3 variants; H3.3, antibody CS207327; H3.1/2, antibody ABE154. Note: * *p* < 0.05; ** *p* < 0.005; Mann–Whitney *U* test related to LGG. (**C**) Western blotting assays in surgical tumor resections of the second cohort depicted as in (**B**). Note: * *p* < 0.05; ** *p* < 0.005; Mann–Whitney *U* test related to grade II gliomas. (**D**) Ratio of the H3.3 and H3.1/H3.2 values from both cohorts.

To elucidate the potential role of H3.3 in GB malignancy, we compared the gene expression levels of *H3-3A* and *H3-3B* between adult GBs (grade IV) and LGG (grades II and III) using the RNA-seq-based transcriptomics datasets contained on the GDC portal (hereinafter referred to as TCGA datasets). We found that the *H3-3A* gene was significantly upregulated in GB compared to LGG (log_2_ fold change = 1.7, adjusted *p*-value < 10^−285^), whereas the *H3-3B* transcript was downregulated (Figure 1A, log_2_ fold change = −0.4, adjusted *p*-value = 4.8 × 10^−16^). This downregulation was confirmed in the REMBRANDT cohort in which the Affymetrix U133 Plus 2.0 microarray platform was used for gene expression profiling (log_2_ fold change = −0.5, adjusted *p*-value = 3.4 × 10^−15^, with the best aligned probe set into the *H3-3B* gene, 211998_at; however, no reliable probe set was available for *H3-3A*). According to TCGA datasets, the average expression levels of *H3-3B* were superior to *H3-3A* in all the gliomas (~20-fold, Figure 1A), therefore we predicted a net decrease in the H3.3 protein in GB after inferring the combined contributions of both transcripts to the protein levels.

To confirm such protein reduction, we examined the H3.3 levels in Western blotting assays in surgical tumor resections from adult patients (Table S1) who had been diagnosed primarily using histological criteria with astrocytoma, oligodendroglioma, either diffuse (grade II: AD and ODD) or anaplastic (grade III: AA and ODA), or GB (grade IV). After normalization with total histone H3, we identified a significant decrease in the protein levels in GB compared to LGG (Figure 1B). We excluded a potential interference of the testis hominid-specific H3.5 variant in our measurements of the H3.3 levels (as their primary sequences possessed 96% homology) by demonstrating that the *H3-5* gene (aka *H3F3C*) was not ectopically activated in gliomas (Figure S1). We validated the decrease in H3.3 in GB compared to low-grade gliomas in the second set of gliomas that enabled the distinction between grade II and III subtypes (Figure 1C).

In parallel, we also explored the protein levels of the canonical variants H3.1/H3.2. In contrast to H3.3, in high-grade gliomas, these protein levels were notably augmented compared to grade II gliomas in both cohorts (Figure 1B,C). Similar results were obtained using an alternative second set of antibodies in the same samples (Figure S2A). Moreover, the differences in the histone H3 variants between grade II and IV gliomas were not due to the altered levels of total histone H3, which were relatively stable across samples of different histological grades compared to other housekeeping proteins in our collection of samples (Figure S2B). Expressing our results as ratios between H3.3 and canonical H3, we determined that H3.3 was largely predominant in grade II gliomas, which shifted towards H3.1/H3.2 in more aggressive gliomas (Figure 1D and Figure S2C). Raw blots are shown in Figure S2D,E.

**Figure 2:**
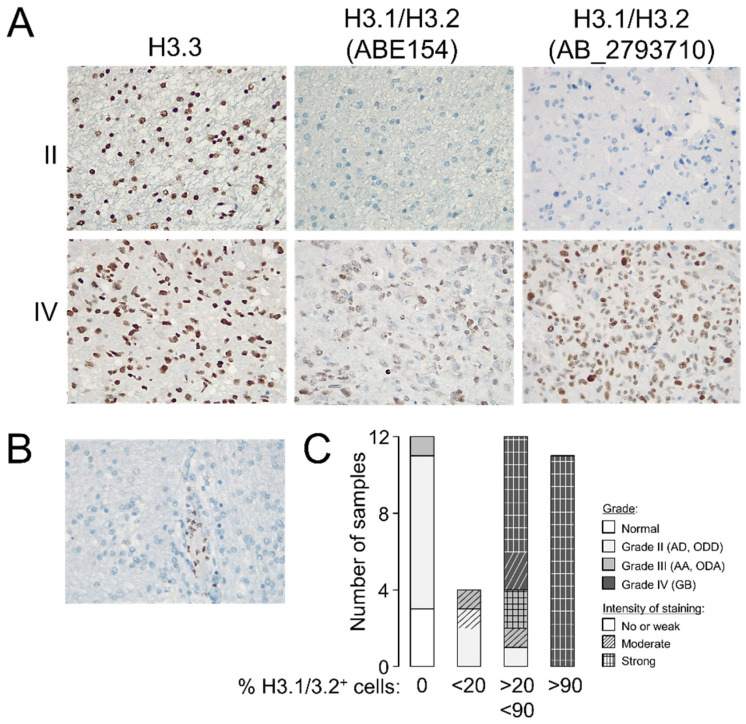
Staining patterns of the histone H3 variants in glioma. (**A**) Representative immunohistochemistry images in grade II and IV gliomas showing the staining patterns of H3.3 and H3.1/H3.2 in the tumoral core (magnification 20×). (**B**) Example of a negative sample for H3.1/H3.2 staining showing positive labeling in leukocytes (magnification 20×). (**C**) Classification of the samples according to the number of positive cells for H3.1/H3.2 staining; histological grade refers to the morphological features observed on the slide used in the immunohistochemistry assay. Note: AD, diffuse astrocytoma; ODD, diffuse oligodendroglioma; AA, anaplastic astrocytoma; ODA, anaplastic oligodendroglioma; GB, glioblastoma.

To validate the potential utility of examining the levels of H3.3 and H3.1/H3.2 at anatomical pathology departments, we performed immunohistochemistry assays in formalin-fixed tumors (*n* = 37, Table S1). Whereas the differences in the H3.3 staining pattern between gliomas of different grades were not obvious to an expert pathologist, H3.1/H3.2 showed a distinctive pattern in GB that included recognizable high-grade cancerous cells (Figure 2A) but without replacing the nuclear distribution of H3.3 (Figure S3). Contrary to the H3.3 expression, null expression of the canonical variants was observed in two thirds of the examined grade II gliomas (Figure 2A,C), in which staining was apparently confined to nontumoral cells such as leukocytes (Figure 2B), which might explain some of the signals observed in the Western blotting analysis. The few available samples of grade III gliomas exhibited a less clear distinction with a trend towards positive staining of H3.1/H3.2^+^ which was in general agreement with the Western blotting results (Figure 1D). Therefore, we demonstrated that the protein levels of the canonical histone H3 variants varied across gliomas with different levels of aggressiveness, and their immunodetection (either by Western blotting or immunohistochemistry assays) might differentiate between low- and high-grade gliomas in diagnosis.

### 3.2. Expression of the Histone H3 Variants Can Be Associated with Clinical Parameters That Define the Degree of Glioma Aggressiveness

**Figure 3:**
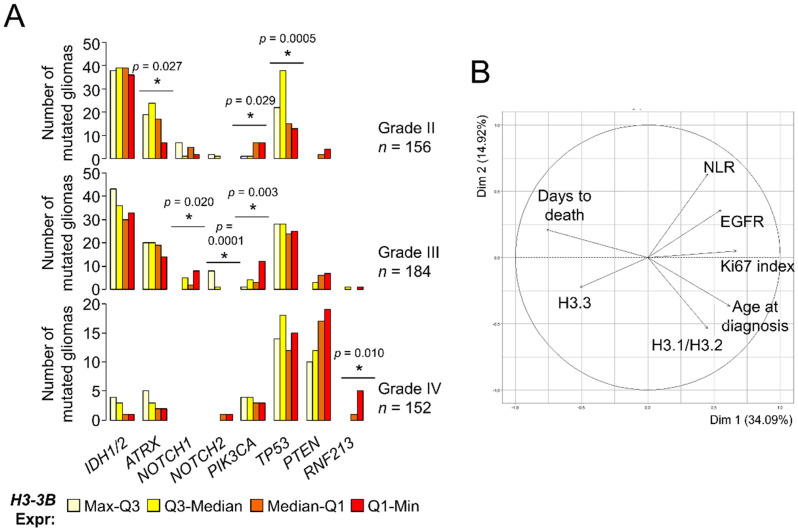
Levels of the histone H3 variants are associated with clinical parameters of gliomas. (**A**) Number of mutated gliomas based upon TCGA database information. Gliomas were classified in quartiles of *H3-3B* mRNA expression. The number of mutated gliomas across the quartile groups was compared to a random distribution. Note: * *p* < 0.05, *χ*^2^ d.f. = 3. (**B**) Clustering of parameters (variables) in two dimensions according to PCA. The projection of the arrows indicates the dominance (direction) and degree of correlation (length) of the represented dimensions (principal components). Circle, maximum correlation.

The reduction of histone H3.3 prompted us to investigate whether its levels constituted a survival predictor in gliomas. A previous work reported the association between *H3-3B* expression and long survival within GB [23] using external information from an independent cohort [30], but we were unable to confirm this point using TCGA datasets (logrank *p*-value = 0.57). Expectedly, *H3-3B* expression was a survival predictor when considering all gliomas (Figure S4A) as the levels of this transcript were differentially expressed between histological grades (Figure 1A). Because molecular signatures are key to define glioma subtypes with different outcomes and response to therapy [31,32], we investigated the H3.3 levels in the context of other molecular alterations with clinical relevance. According to TCGA, grade II gliomas showing high expression levels of *H3-3B* tend to accumulate more somatic mutations in the *ATRX* and *TP53* genes (Figure 3A), which are most frequently found in astrocytomas with IDH mutations [33]. In contrast, grade II and III gliomas showing low expression levels of this mRNA were more frequently mutated in the *PIK3CA* gene (Figure 3A), which may be plausibly linked to poor prognosis [34]. Furthermore, distribution of the *NOTCH1* and *NOTCH2* mutations across grade III gliomas was also significantly dependent on *H3-3B* expression. Lastly, the comparison between GBs with different levels of H3.3 expression did not reveal any potential association with relevant clinical markers, except for *RNF213* (Figure 3A), for which mutations are linked to cerebrovascular pathologies [35] and are unfavorable in other cancers [36,37]. These results suggested that gliomas with different expression levels of *H3-3B* were more likely linked with distinctive patterns of somatic mutations that can be useful for prognosis and deserve further exploration in a large cohort.

Using the molecular information of our cohorts, we investigated the potential association of histone H3 protein expression with the IDH1R132H mutation, loss of heterozygosity (LOH)1p/19q, EGFRvIII mutation, *EGFR* upregulation and hypermethylation of the *MGMT* promoter (see Materials and Methods and the Supplementary Information for further details). Overall, the samples with the lowest expression of H3.3 tended to be wild-type for *IDH1* mutation and LOH 1p/19q, with unmethylated *MGMT* promoter (*p*-value < 0.05, χ^2^ d.f. = 9) (Figure S4B). Apart from the molecular parameters, we also inspected other potential correlates of the histone H3 levels such as age at diagnosis, survival after diagnosis (days to death), proliferative index (% of Ki67^+^ cells) and preoperative neutrophil/lymphocyte ratio (NLR), as it was suggested that high NLR values are linked to special aggressive GBs [38,39]. Of these, age and proliferative index were significantly correlated with H3.3 and H3.1/H3.2 in opposite directions, respectively (Figure S4C). No clear patterns were observed considering the location of the tumor (Figure S4D). Next, we examined the contribution of all these clinical and molecular parameters in a single multivariate analysis. To this aim, we imputed the missing values (i.e., by replacing the values lacking in the diagnosis by plausible values that were predicted according to the overall variance across the parameters [40]) prior to performing PCA (see Materials and Methods). In general terms, this PCA showed that the H3.1/H3.2 levels were more associated with age at diagnosis, followed by the proliferative index (characteristic of highly aggressive gliomas). These parameters, together with *EGFR* mRNA expression and NLR, were opposed to the H3.3 levels, which were relatively close to survival (days to death) (Figure 3B). This result confirmed the differential association of the histone H3 variants with gliomas of different degrees of malignancy, which were largely concordant with the histological classification.

### 3.3. Genes Encoding the Canonical Histone H3 Variants Showed a Complex Induction Pattern in Glioblastomas

**Figure 4:**
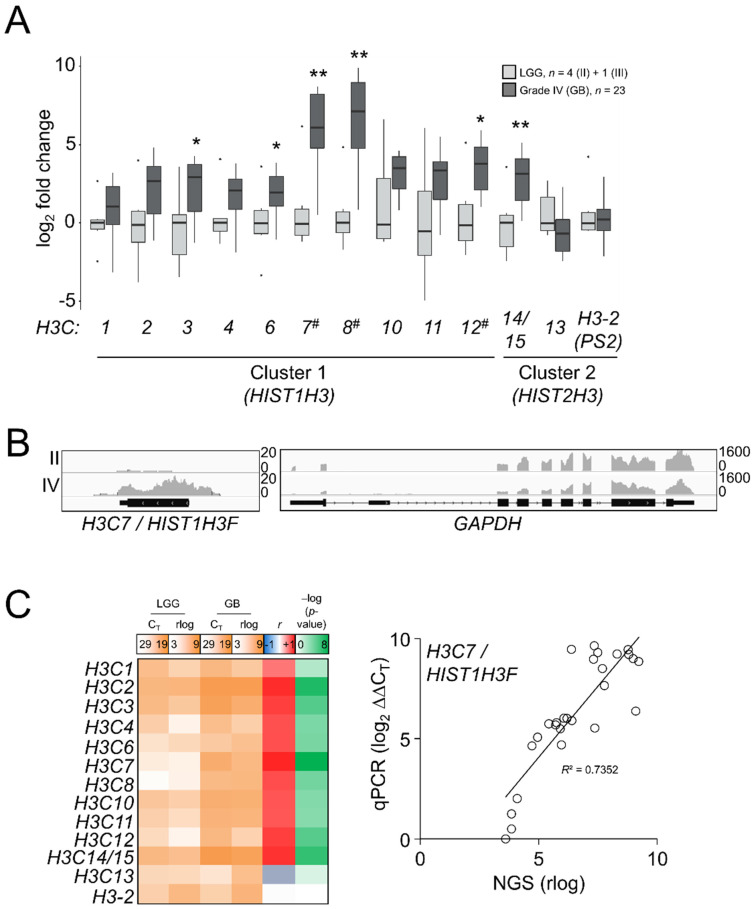
Differential mRNA expression of the canonical H3 variant-encoding genes across gliomas of different grades. (**A**) RT-qPCR assays in the samples of the first cohort for the canonical histone H3 genes. *H3.X* and *H3.Y* were not expressed and they are not depicted. *X*-axis numbering refers to each *H3C* gene (e.g., *H3C1* as *1*). Note: * *p* < 0.05; ** *p* < 0.005; Mann–Whitney *U* test related to the lowest grade; #, *p* < 0.05; Kendall’s rank correlation between mRNA and the H3.1/H3.2 protein variations. LGG, lower-grade glioma; GB, glioblastoma. (**B**) Reads alignments across the *H3C7* and *GAPDH* loci in a grade II and a grade IV samples. *Y*-axis, number of counts. (**C**) Mean gene expression (qPCR C_T_ values and rlog sequencing counts) in LGG and GB, plus Pearson correlation coefficients (*r*) and corresponding –log (*p*-values) for each histone H3 mRNA between RT-qPCR (qPCR) and RNA-seq (NGS); right panel, the correlation for the *H3C7* gene as an example.

As there was an apparent reactivation of H3.1/H3.2 expression in the most aggressive gliomas, we interrogated whether such induction was associated with transcriptional patterns for the canonical H3 genes: *H3C1-4* (*HIST1H3A-D*), *H3C6-8* (*HIST1H3E-G*), *H3C10-12* (*HIST1H3H-J*), corresponding to H3.1, and *H3C13-15* (*HIST2H3D/C/A*) and the potentially expressed pseudogene *HIST2H3PS2* named as *H3-2* (https://www.ensembl.org/Homo_sapiens/Gene/Summary?db=core;g=ENSG00000273213;r=1:143894544-143905966, accessed on 10 September 2021), corresponding to H3.2. We also included in the analysis the unrelated variants *H3.X* and *H3.Y*, which were not expressed in our samples. This analysis might discriminate which genes could be the most responsible for differential protein expression in high-grade gliomas. In contrast to the H3.3-encoding genes, replication-dependent histone genes are transcribed into nonpolyadenylated mRNAs [41]; thus, TCGA and the REMBRANDT datasets generated after poly-RNA selection [42,43,44,45] were not appropriate to investigate the gene expression variations of the H3.1 and H3.2 transcripts (Figure S5A). Instead, we conducted a survey of the canonical histone H3 transcripts in our samples by qPCR assays after retrotranscription using random hexamer primers. After checking the specificity of the primers (Figure S5B), we observed general induction of the majority of the canonical histone H3 genes in GB compared to LGG, with the most striking results for the *H3C7*, *H3C8* and *H3C12* transcripts (Figure 4A), the levels whereof significantly correlated with the abundance of the H3.1/H3.2 protein (*p* < 0.01, Kendall’s rank correlation). To discard a potential effect related to the selection of our housekeeping genes in these assays, we conducted an NGS experiment in some of the samples used in the RT-qPCR assays by using the RNA-exome approach that allowed the sequencing of histone genes. Apart from showing higher expression levels than mRNA-seq and the Affymetrix-based platform (Figure S5A), this assay also confirmed the validity of the RT-qPCR results for most of the transcripts (Figure 4B,C). Thus, we demonstrated that variations in the protein levels of the canonical histone H3 variants across gliomas with different levels of aggressiveness could be at least partly explained by transcriptional alterations.

**Figure 5:**
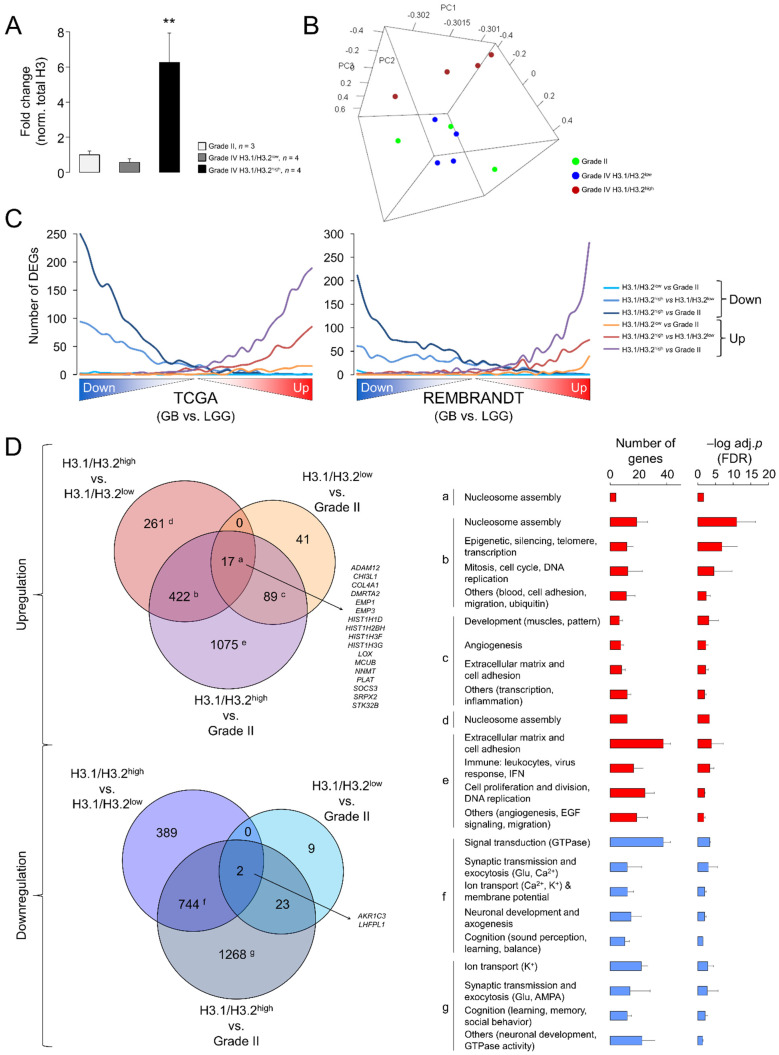
Differential gene expression between glioblastomas expressing low and high levels of the canonical H3 proteins. (**A**) H3.1/H3.2 protein levels in the selected samples for the differential expression analysis, classified in three groups: grade II gliomas and GB expressing low and high levels of protein (H3.1/H3.2^low^ and H3.1/H3.2^high^, respectively). The data are expressed as the means ± SEM. Note: ** *p* < 0.005; Mann–Whitney U test related to grade II gliomas. (**B**) Tridimensional PCA of the samples used in the differential expression analysis considering their whole transcriptomes. (**C**) Distribution of differentially expressed genes (DEGs) obtained in the three pairwise comparisons (H3.1/H3.2^high^ vs. H3.1/H3.2^low^, H3.1/H3.2^high^ vs. grade II gliomas and H3.1/H3.2^low^ vs. grade II gliomas) across the whole GB transcriptomes from TCGA (left) and the REMBRANDT (right) cohorts. These transcriptomes were ranked according to the significance and direction of the gene expression change compared to lower-grade gliomas and divided into bins of 500 genes. The number of down- and upregulated genes from our lists was counted in each bin. (**D**) Venn diagrams showing the overlaps between the lists of DEG (adj. *p*-value < 0.05) from the following comparisons: H3.1/H3.2^low^ vs. grade II gliomas, H3.1/H3.2^high^ vs. grade II gliomas, H3.1/H3.2^high^ vs. H3.1/H3.2^low^. Letters refer to the significantly enriched GO terms (FDR < 0.05, DAVID) for each subset of genes represented as the number of genes and –log-adjusted *p*-value (mean ± SD); red and blue bars, upregulation and downregulation, respectively.

Despite the significant differences related to LGG, the results of the Western blotting (Figure 1) and RT-qPCR assays (Figure 4) evidenced a large variability in the expression levels of the canonical H3 variants in GB. To infer the putative functional implications of this heterogeneity, we compared the transcriptome profiles between GBs expressing the highest and lowest levels of the H3.1/H3.2 proteins with the associated RNA-seq data (hereinafter referred to as H3.1/H3.2^high^ and H3.1/H3.2^low^, respectively) (Figure 5A). Nearly two thirds of the differentially expressed genes (DEGs) were downregulated in H3.1/H3.2^high^ compared to H3.1/H3.2^low^ GBs (Table S3). Of note, among the most upregulated genes, we found several histone-coding genes, including histone H3 (*H3C7*, *H3C12* and other 12 genes), confirming that differential protein expression of the canonical H3 variants across gliomas was partially explained by differential gene expression. In addition, we observed upregulation of the rest of the constituents of the nucleosome, i.e., of H2A (14 DEGs), H2B (15 DEGs) and H4 (eight DEGs), and of the linker histone H1 (four DEGs) (Table S3). As we also observed differential expression of well-known cancer-related genes, such as upregulation of the *MYC* oncogene in the H3.1/H3.2^high^ samples, we asked whether the expression of the canonical histone H3 proteins might be associated with different degrees of glioma aggressiveness. To this aim, we contrasted the gene expression profiles of grade II gliomas (characterized by low levels of H3.1/H3.2) with those of H3.1/H3.2^high^ and H3.1/H3.2^low^ GBs, resulting in the retrieval of >3500 DEGs (H3.1/H3.2^high^ GB vs. grade II gliomas) and <200 DEGs (H3.1/H3.2^low^ GB vs. grade II gliomas) (Table S3). This observation indicated that, according to the levels of H3.1/H3.2, H3.1/H3.2^low^ GBs were transcriptionally more similar to low-grade gliomas than H3.1/H3.2^high^ GBs, as confirmed by PCA of the whole transcriptomes (Figure 5B). Next, we plotted the distribution of DEGs of the three pairwise comparisons (H3.1/H3.2^high^ vs. H3.1/H3.2^low^, H3.1/H3.2^high^ vs. grade II gliomas and H3.1/H3.2^low^ vs. grade II gliomas) across the whole GB transcriptomes of TCGA and the REMBRANDT repositories, in which transcripts were ordered according to their differential expression significance related to the LGG profiles of the same databases. As expected, DEGs in H3.1/H3.2^high^ GBs compared to grade II gliomas reproduced the differential expression between GB and LGG of both cohorts in the same direction of change, followed by DEGs in H3.1/H3.2^high^ compared to H3.1/H3.2^low^ GBs, and to a much lesser extent by DEGs in H3.1/H3.2^low^ GBs compared to grade II gliomas (Figure 5C). This behavior was reproduced by the canonical histone H3 genes as they were increasingly significant across the pairwise comparisons (Figure S5C). Altogether, these results indicated that H3.1/H3.2^low^ GBs might represent a transcriptional intermediate state that was relatively close to low-grade gliomas.

To determine the functions that were associated with these DEGs, we conducted a Gene Ontology (GO) analysis that revealed enrichment of genes related to the “Nucleosome assembly” function (mainly due to the upregulation of the histone-coding genes) in the common upregulated genes in the three pairwise comparisons (Figure 5D, adjusted *p*-value < 0.05). This function was also enriched in the common DEGs between H3.1/H3.2^high^ and the other two groups of gliomas that were accompanied by GO terms related with DNA replication and mitosis, suggesting that upregulation of components of the nucleosome and chromatin might be associated with high rates of cell division in H3.1/H3.2^high^ GBs. This upregulation paralleled downregulation of the genes with neuron-related functions, such as synaptic transmission, ion transport, neuronal development and cognitive processes (Figure 5D), probably reflecting a more prominent reduction of functional neurons within the H3.1/H3.2^high^ tumors. Altogether, these results suggested a special malignancy of H3.1/H3.2^high^ GBs compared to H3.1/H3.2^low^ GBs.

## 4. Discussion

In this work, we found for the first time variations in the levels of histone H3 variants that can be observed directly in highly heterogeneous surgical resections with differential aggressiveness [2] as a means to improve diagnosis using routine clinical techniques. In general, grade II gliomas expressed higher levels of H3.3 and weak levels of H3.1/H3.2 compared to GB. Within grade III gliomas, astrocytomas more closely resembled GB compared to oligodendroglioma (Figure S2C), although it will require a large number of samples to confirm such a distinction. Therefore, measuring the levels of these histone variants may facilitate further refinement of the distinction between low-grade (II) and high-grade (III, IV) gliomas, which can be misclassified during diagnosis as a result of interobserver and interinstitutional variations [46]. This refinement is crucial for applying the most suitable treatment as the combined radiotherapy and chemotherapy frequently used in GB can be particularly aggressive for elderly patients with associated comorbidities. Nonetheless, the trend in clinical diagnosis is to incorporate molecular signatures [3] (by defining combinations of alterations in *IDH1*, *EGFR*, *TP53*, *PDGFRA*, *PTEN*, *TERT*, *MGMT*, etc.) to improve the personalized counseling of the patients as the assays are becoming more affordable and technically accessible for clinical services [47]. Following this line of argument, our own study identified potential biomarkers of good prognosis (*ATRX* and others) that were more apparently frequent in gliomas with higher levels of *H3-3B*/H3.3, whereas some biomarkers of poor prognosis (*PIK3CA*) were more strongly associated with gliomas with low levels of *H3-3B*, suggesting that the H3.3 levels might be useful for the prognosis of low-grade tumors. Although these observations should be confirmed in additional cohorts, this was in agreement with the reduction of gene expression levels for H3.3 in GSC preparations that led to their perpetuation in a proliferative and immature state [23]. For its part, high levels of H3.1/H3.2 were more associated with age at diagnosis, proliferation index and *EGFR* mRNA alterations which were features of aggressive GB. These results indicate that the histone H3 variants should be further explored as part of the molecular criteria to classify gliomas of different clinical outcomes.

During this study, we also established the most appropriate methods to detect such variations: histone H3 variants levels can be conveniently quantified by Western blotting, whereas H3.1/H3.2 from cancer cells and the surrounding cells can be discriminated in immunohistochemistry assays. Whether the number of positive cells can be associated with aggressiveness, as predicted by the transcriptomics analysis of H3.1/H3.2^high^ and H3.1/H3.2^low^ GBs, and, as a consequence, have a significant impact on the overall survival remains to be investigated in a large cohort of patients. Note that we need to establish standard criteria to adequately score the immunostainings according to the number of positive cells as this is the first time that the H3.1/H3.2 markers have been examined in glioma slices. In the case of transcript levels, to the best of our knowledge, only a single study has attempted to survey the expression of H3.1 in gliomas, more precisely the *H3C12*/*HIST1H3J* mRNA, which concluded that this transcript was significantly upregulated in high-grade gliomas [48], in agreement with our results. Thus, our study is the first report to screen the expression levels of the genes contained in clusters 1 and 2 encoding canonical histone H3 in gliomas. However, we found that protein levels were more reliable and easier to interpret than complex gene expression patterns in the special case of histones, in which protein expression is the final convergence of multiple transcriptionally deregulated genes that may show heterogeneous expression patterns. Moreover, we cannot exclude alterations in protein synthesis and turnover. We should also consider that half-lives of histones, which can be especially long in the non-dividing cells [49,50] within tumoral resections, may mask the potential impact of gene expression changes at the level of protein.

In any case, histone H3 dysfunction in adult gliomas was largely dependent on expression levels rather than somatic mutations. In addition to the lack of consistent mutations affecting the histone H3 genes in TCGA database (Table S4), we should consider in the light of our results that the induction of multiple canonical histone H3 genes in GB impose that rare mutations may only affect a small fraction of the total H3.1/H3.2 proteins. In contrast, mutations in the *H3-3A* gene are highly relevant in pediatric GB but they have a dominant-negative effect, while the reduction of the protein levels in adult *H3-3A* wild-type GB indicated a loss of function [23,51]; in the few adult cases with the mutated gene [52], it will be interesting to elucidate whether both mechanisms of H3.3 dysfunction coexist. Therefore, the impact of such variations should be clarified. For instance, H3.3 reduction may destabilize DAXX-containing complexes to promote tumorigenesis [53,54]. These complexes can contain PTEN, which controls oncogenic expression in GB cells through the deposition of H3.3 into the chromatin [54]. Whether the reduction of H3.3 may mimic to some extent the effects of the PTEN loss-of-function remains to be examined, although we did not find a link between the frequency of PTEN mutation and *H3-3B* expression in glioma (Figure 3A). Hence, H3.3 reduction may allow perpetuation of GSCs in accordance with the role of this variant in controlling the proliferation and differentiation of neural stem cells during brain development [55]. H3.3 dynamics are associated with chromatin integrity [56], which can be especially relevant to explain the genomic instability of cancer processes. In contrast, the roles of H3.1 and H3.2 are more obscure but may have distinct roles in chromatin organization [57].

Because the levels of these replication-dependent variants were associated with proliferation and mitosis (Figure S4C and Figure 5D), their induction might be linked to the characteristic cell proliferative activity of high-grade gliomas. As H3.3 is the main histone H3 variant in the mostly postmitotic adult brain [50], we may speculate that there is an exchange of histone H3 variants in higher-grade gliomas favoring incorporation of the canonical variants into the chromatin to the detriment of H3.3 in the cancer cells that acquire an immature-like state of proliferation and self-renewal. In fact, glioma cells share common pathways with normal neural progenitor cells during cancer progression [58]. However, we did not find evidence of H3.1/H3.2 actually replacing H3.3 occupancy across the GB chromatin (Figure S3). Further studies will determine whether these histone H3 variants confer different functions to modulate the expression of oncogenes and/or tumor suppressor genes.

## 5. Conclusions

We propose that levels of histone H3 proteins can help in refining the diagnosis of low- and high-grade gliomas (linked to higher levels of the H3.3 and H3.1/H3.2 variants, respectively), and potentially contribute to the classification of different subtypes of gliomas in combination with other molecular parameters. In addition, we provide initial evidence justifying further exploration of the involvement of the histone H3 variants in epigenetic dysregulation of glioma cells and cancer malignancy, which may be extended to other histones (i.e., H2A, H2B and H4).

## Data Availability

In-house datasets generated and analyzed during the current study are available in the GEO repository under accession number GSE185861.

## Supplementary Materials

The following are available online at https://www.mdpi.com/article/10.3390/cancers13215261/s1, Figure S1: No apparent reactivation of the *H3-5* gene in glioblastoma, Figure S2: Western blots of the histone H3 variants in gliomas, Figure S3: Colocalization patterns of the histone H3 variants in glioblastoma cells, Figure S4: Additional information regarding the expression of the canonical histone H3 variants, Figure S5: Additional correlative analysis between clinical parameters and the histone H3 variants, Table S1: Clinical characteristics of the cohorts of patients used in the study, Table S2: Sequences of the primers used in this study, Table S3: RNA-seq results of the gliomas expressing different levels of the H3.1/H3.2 protein, Table S4: Somatic mutations in the histone H3-encoding genes (TCGA database).
