# Supplementary material for: Potential Diagnostic Value of the Differential Expression of Histone H3 Variants between Low- and High-Grade Gliomas
Source: Cancers (Basel). 2021 Oct 20;13(21):5261. doi: 10.3390/cancers13215261 (PMC8582563; doi:10.3390/cancers13215261)
Supplement: Supplementary file 1 [file cancers-13-05261-s001.zip › Supplementary Information editable.pdf]

cancers MDPI

Supplementary Files

Potential Diagnostic Value of the Differential Expression of Histone H3 Variants between Low- and High-Grade Gliomas

Irati Hervás-Corpión et al.

1. Supplementary Methods

1.1. Supplementary Molecular Diagnosis

To determine hypermethylation of the *MGMT* promoter, we obtained gDNA from protein extracts after treatment with proteinase K (20 mg/mL, Thermo Fisher, Madrid, Spain) for 2 h at 55 °C followed by extraction with phenol-chloroform-isoamyl alcohol (Sigma-Aldrich, Darmstadt, Germany) and precipitation with sodium acetate and ethanol. A total of 250 ng of gDNA was subjected to bisulfite conversion using the Premium Bisulfite Kit (Diagenode, Seraing, Belgium) according to the manufacturer's instructions. Subsequent PCR was carried out with BioTaq polymerase (Bioline, Barcelona, Spain) using primers specific for methylated and unmethylated *MGMT* promoter (Table S2), as previously reported [1]. The PCR cycling conditions were as follows: 94 °C for 3 min, 25 cycles of 94 °C for 20 s, 60 °C for 20 s, 72 °C for 30 s and a final step at 72°C for 5 min in a GeneAmp PCR System 2700 thermocycler (Applied Biosystems, Thermo Fisher, Madrid, Spain). gDNA from normal peripheral blood mononuclear cells (PBMCs) obtained after density gradient centrifugation using Histopaque-1077 (Sigma-Aldrich, Darmstadt, Germany) was used as a negative control for methylated alleles of *MGMT*. Each PCR was loaded on a 2% agarose gel, stained with RedSafe (iNtRON Biotechnology, Sangdaewon-Dong, South Korea) and visualized under UV illumination.

For 1p/19q codeletion analysis, glioma resection and peripheral blood from each patient were used for gDNA extraction using the QIAamp DNA Mini Kit (Qiagen, Hilden, Germany) according to the manufacturer´s recommendations. The LOH in 1p/19q was determined by PCR in a T100 Thermal Cycler (Bio-Rad, Madrid, Spain) by using 4 microsatellite markers for the 1p chromosome (D1S199, D1S508, D1S2696, D1S2734) and 3 markers for the 19q chromosome (D19S898, D19S412, D19S596) comparing the results between the tumoural tissue and non-tumoural whole blood as previously reported [2-4]; we concluded that there was LOH when the ratios between blood / tumour was < 0.80 of the height of the peaks of both alleles for each marker. The sequences of these markers are included in Table S2. The PCR cycling conditions were as follows: 98 °C for 15 min and 30 cycles of 98 °Cfor 15 s, 57 °C for 15 s, 72 °C for 20 s and a final step at 72 °C for 30 min. Finally, PCR products were separated by capillary electrophoresis in an ABI PRISM 3100 genetic analyzer (Applied Biosystems, Madrid, Spain) and analyzed using GeneMapper software (v3.7).

To detect EGFRvIII, we set up qPCR assays from cDNA using specific primers spanning exon 1 and 8 of *EGFR* gene (Table S2) following the procedure described in the main text.

1.2. Plasmids and Transient Transfection Assays

We designed pairs of primers to clone the full-length cDNAs of *H3-3A*, *H3-3B* and *H3-5* for the expression of H3.3 and H3.5 (Table S2). As the primers were not able to distinguish between *H3-3B* and *H3-5*, we amplified *H3-3B* from PBMC cDNA, taking advantage of the null expression of *H3-5* mRNA in these cells, and *H3-5* from PBMC gDNA, taking advantage of the intron that was only present in *H3-3B*. All reactions were performed with Q5 High Fidelity DNA polymerase (New England Biolabs, Ipswich, USA) in a GeneAmp PCR System 2700 thermocycler (Applied Biosystems, Thermo Fisher, Madrid,

Spain). The PCR cycling conditions were 98 °C for 30 s and 35 cycles of 98 °C for 10 s, 62 °C for 30 s, 72 °C for 30 s and a final step at 72 °C for 5 min. The forward primers contained the restriction sites for Sal I (*H3-3A*) or EcoR V (*H3-3B*/*H3-5*), and the reverse primers contained the restriction site for Sac II, which enabled the cloning of the resulting PCR in the plasmid pIRES2-EGFP (Clontech, Mountain View, CA, USA), which was previously modified to contain a Kozak sequence and 2 HA-tags in tandem between the EcoR I and Sal I sites of the multiple cloning site for the expression of fusion proteins. Sanger sequencing (STAB-VIDA service, Portugal) validated the sequences of the inserts.

Plasmid preparations were purified with the Nucleobonds Xtra Midi kit (Mackerey-Nagel, Madrid, Spain) following the manufacturer's instructions, transfected into the HEK293 cell line, cultured the day before in 24-well plates treated with poly-L-lysine (Sigma-Aldrich, Darmstadt, Germany) and maintained in complete medium: DMEM high glucose with sodium pyruvate (Biowest, Barcelona, Spain), 100 U penicillin/100 µg streptomycin/0.29 mg/ml glutamine (Thermo Fisher, Madrid, Spain), and 10% foetal bovine serum (Gibco, Madrid, Spain). Transient transfection was performed using the GenJet In Vitro DNA Transfection reagent according to the manufacturer's recommendations (SignaGen, Frederick, MD, USA). Samples were collected two days after transfection for RT-qPCR analysis.

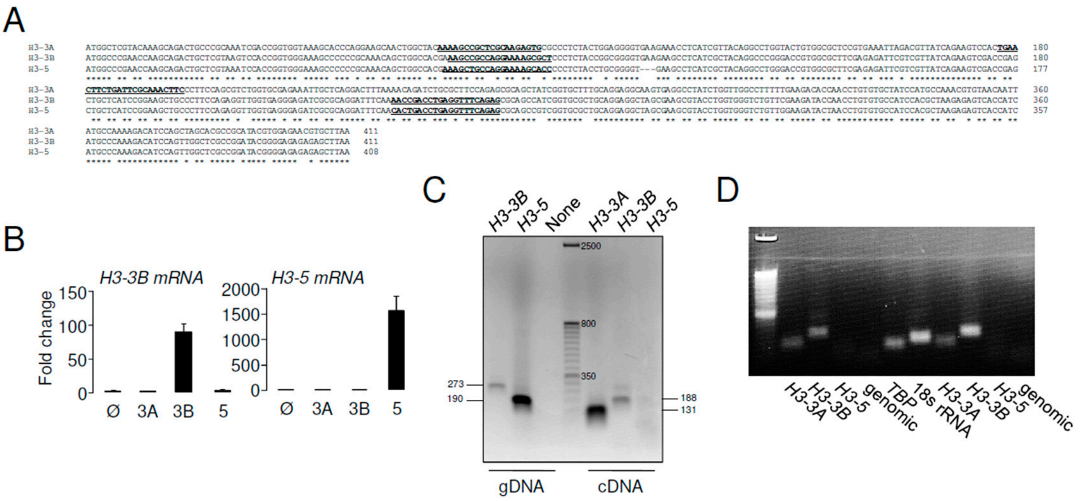

Figure S1. No apparent reactivation of *H3-5* gene in glioblastoma. (**A**) cDNA sequences of *H3-3A*, *H3-3B*, and *H3.5* aligned with the Clustal Omega tool. Primers used in the PCR/qPCR analysis are indicated in underlined bold letters. (**B**) Gene expression analysis of HEK293 cells transiently transfected with different versions of pIRES2-EGP-based plasmids: Ø (empty vector), 3A (*H3-3A*), 3B (*H3-3B*) and 5 (*H3-5*) to validate the specificity of the primers to detect *H3-3B* and *H3-5* mRNAs. (**C**) Gel electrophoresis of PCR products of *H3-3A*, *H3-3B* and *H3-5* in gDNA and cDNA of a representative glioblastoma (GB) sample. None, reaction without primers. Numbers indicate the size of the amplicons, which were different for *H3-3B* in both types of samples due to the presence of a short intron between the primers sequences in the gDNA. (**D**) More examples of PCR products of *H3-3A*, *H3-3B* and *H3-5*, together with intergenic region between *H3-3B* and *UNK* (genomic) as negative control, and the housekeeping genes *TBP* and 18s RNA as positive controls in cDNA of GB samples.

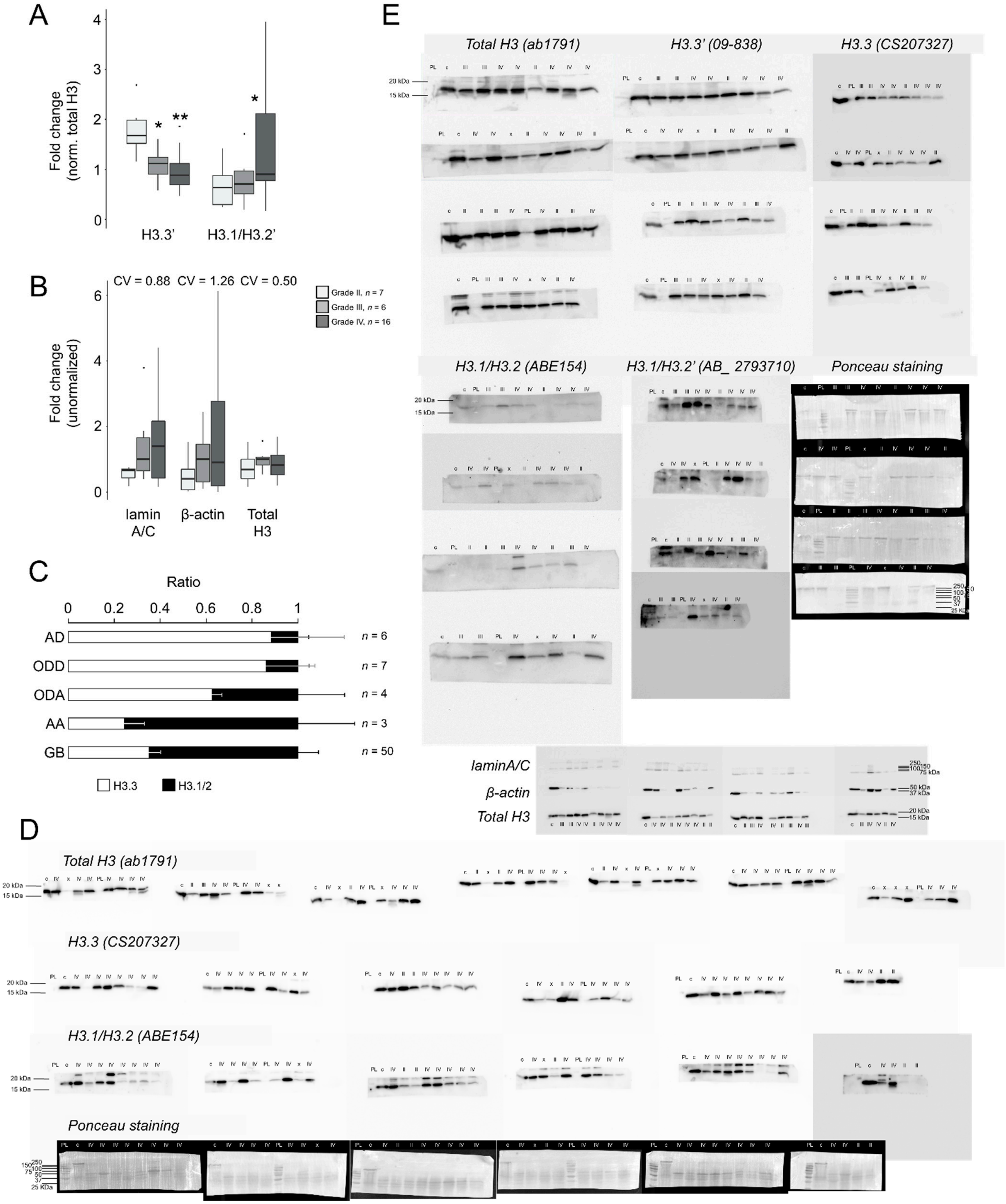

Figure S2. Western blots of histone H3 variants in gliomas. (**A**) Quantification of the Western blotting assays in surgical tumour resections of second cohort, after normalizing with total H3. H3.3′, antibody 09-838; H3.1/2′, antibody AB_2793710. * $p < 0.05$; ** $p < 0.005$; Mann Whitney *U*-test related to grade II. (**B**) The same as in **A** with the following antibodies: laminA/C, 4777; β-actin, A5441; total H3, ab1791. Above the quantifications, the coefficients of variations (CV = SD / mean) are indicated for each detected protein. Histone H3 levels were the less variable across gliomas compared to lamin A/C and β-actin: in the latter cases, variations in the protein levels might be explained by the upregulation of the corresponding mRNAs in GB related to lower-grade gliomas in the TCGA datasets (*LMNA*, $\log_2$ fold change = 0.85, adj. *p*-value = 4.52 x 10^{-46}; *ACTB* $\log_2$ fold change = 0.64, adj. *p*-value = 8.56 × 10^{-49}) and in the REMBRANDT datasets (*LMNA*, 203411_s_at, $\log_2$

fold change = 0.74, adj. p-value = 5.58 × 10^{-34}; *ACTB*, 213867_x_at, $\log_2$ fold change = 0.14, adj. p-value = 1.96 × 10^{-20}). (**C**) Summary of the Western blotting results combining the ratios from our cohorts and differentiating between astrocytomas and oligodendrogliomas. AD, diffuse astrocytoma; ODD, diffuse oligodendroglioma; AA, anaplastic aastrocytoma; ODA, anaplastic oligodendroglioma; GB, glioblastoma. (**D-E**) Raw Western blots detecting the levels of histone H3.3, H3.1/H3.2 and total H3 in the protein extracts from surgical resections of first (**D**) and second cohorts (**E**). Numbers indicate the histological degree, c is the control for normalization between blots, PL the protein ladder (Kaleidoscope, Bio-Rad, Madrid, Spain) and x are the discarded samples for the analysis (low levels of total histone H3, doubtful samples).

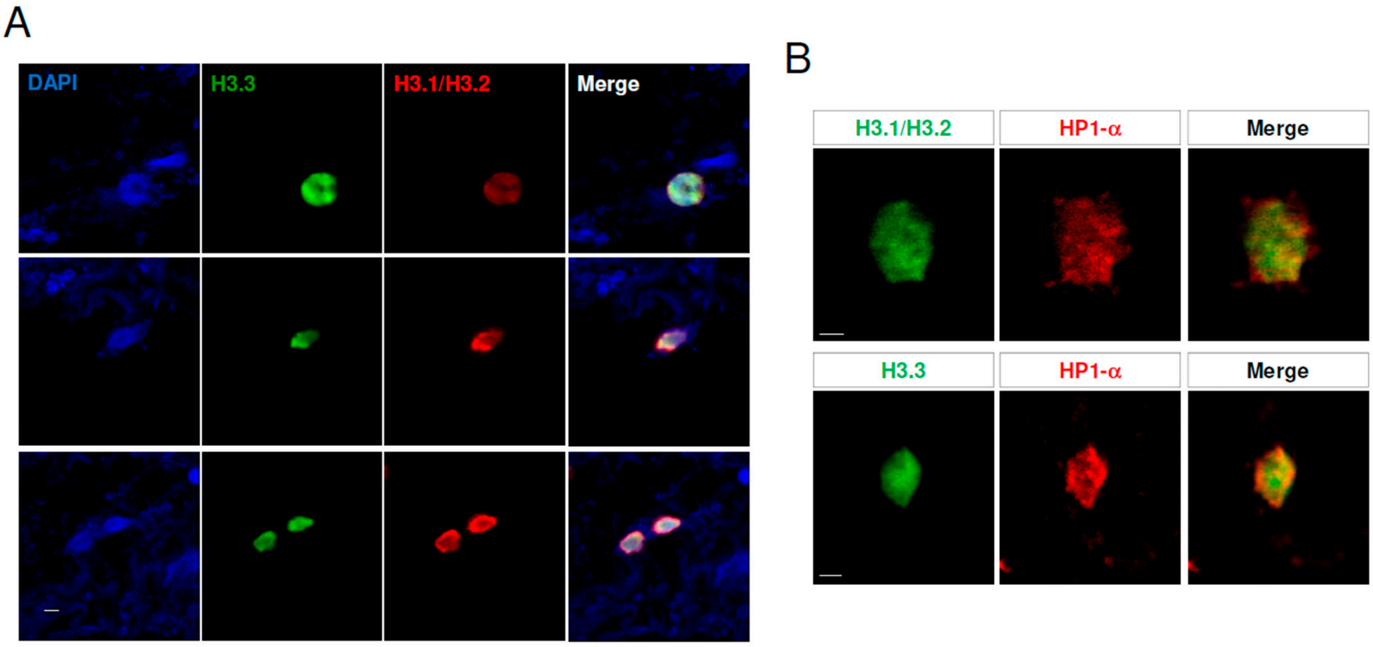

Figure S3. Colocalization patterns of histone H3 variants in glioblastoma cells. (**A**) Representative confocal microscopy images of GB tissue slides. Slides were processed for the immunohistochemical detection of H3.3, H3.1/H3.2 and counter-stained with DAPI to denote nuclei. Scale bar = 20 μm. (**B**) Representative confocal microscopy images of GB tissue slides processed for the immunohistochemical detection of H3.1/H3.2, H3.3 and the heterochromatin marker HP1-α. Histone H3 variants did not show preference towards euchromatin and heterochromatin (characterized by diffuse and punctate patterns, respectively). Scale bar = 10 μm.

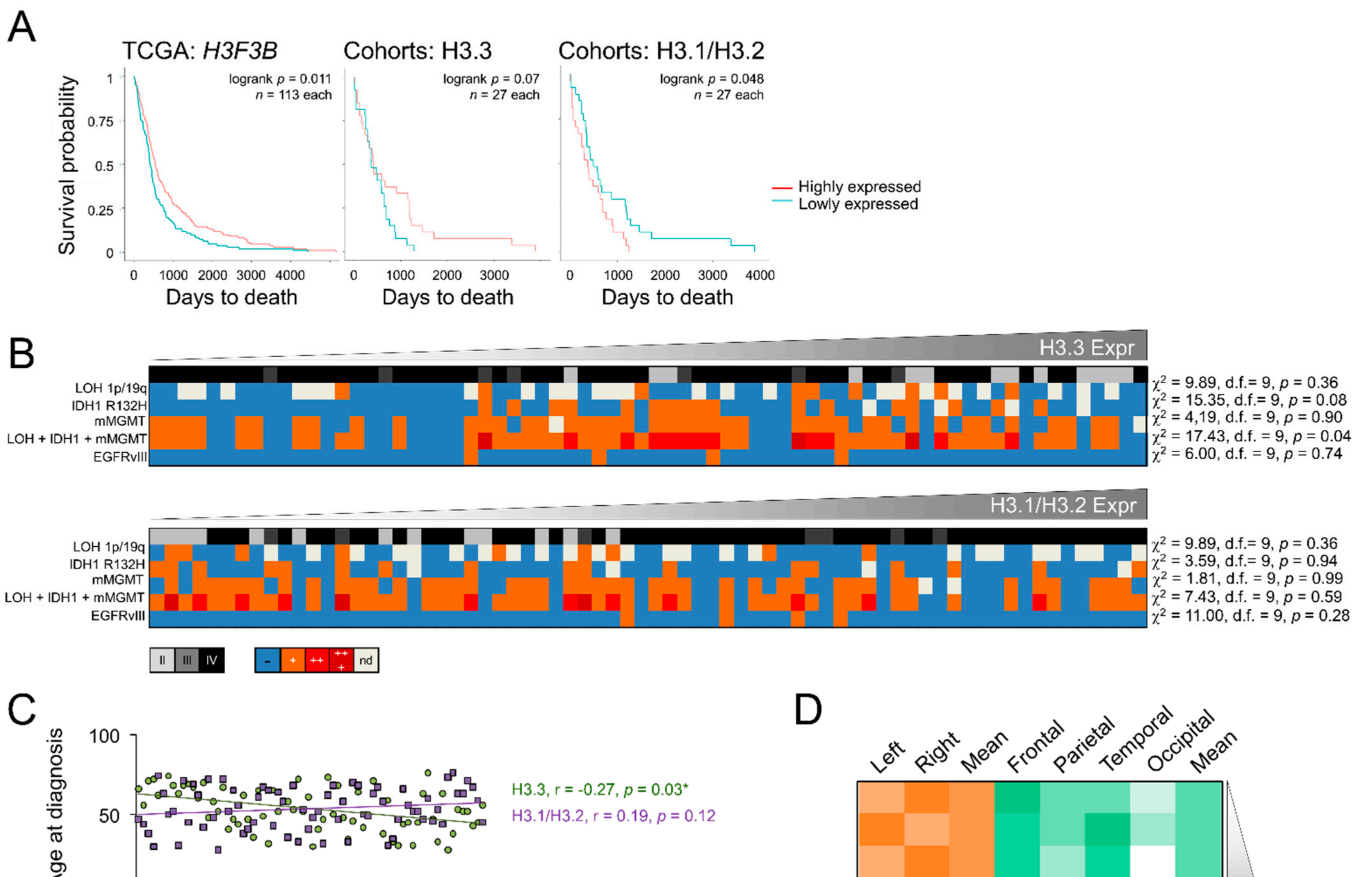

Figure S4. Additional correlative analysis between clinical parameters and histone H3 variants. (**A**) Survival Kaplan-Meier curves between samples showing high and low expression of histone H3 variants: according to the *H3-3B* mRNA levels in all gliomas of the TCGA database (left panel), according to the H3.3 protein levels (normalized CS207327 values to total histone: ab1791) in all gliomas of our cohorts (middle panel), and according to the H3.1/H3.2 protein levels (normalized ABE154 values to total histone: ab1791) in all gliomas of our cohorts (right panel). (**B**) Distribution of the molecular diagnosis parameters (LOH 1p/19q, IDH1 R132H and methylated *MGMT* and EGFRvIII) across the gliomas of our cohorts ranked according to H3.3 or H3.1/H3.2 protein levels. -, negative; +, ++ and +++, positive for 1, 2 and 3 parameters, respectively; nd, not determined/not conclusive. (**C**) Distribution of parameters across the gliomas of our cohorts ranked according to H3.3 or H3.1/H3.2 protein levels, with associated Pearson's correlation coefficients and *p*-values. Only correlations marked with * were significant (p-value < 0.05). (**D**) Number of tumours resected from the indicated hemispheres (left or right) and lobes (frontal, parietal, temporal and occipital); samples located between two lobes were counted in both compartments. No apparent imbalance was detected between samples with different expression levels of histone H3 variants (p-value > 0.05, χ^2 d.f.= 6).

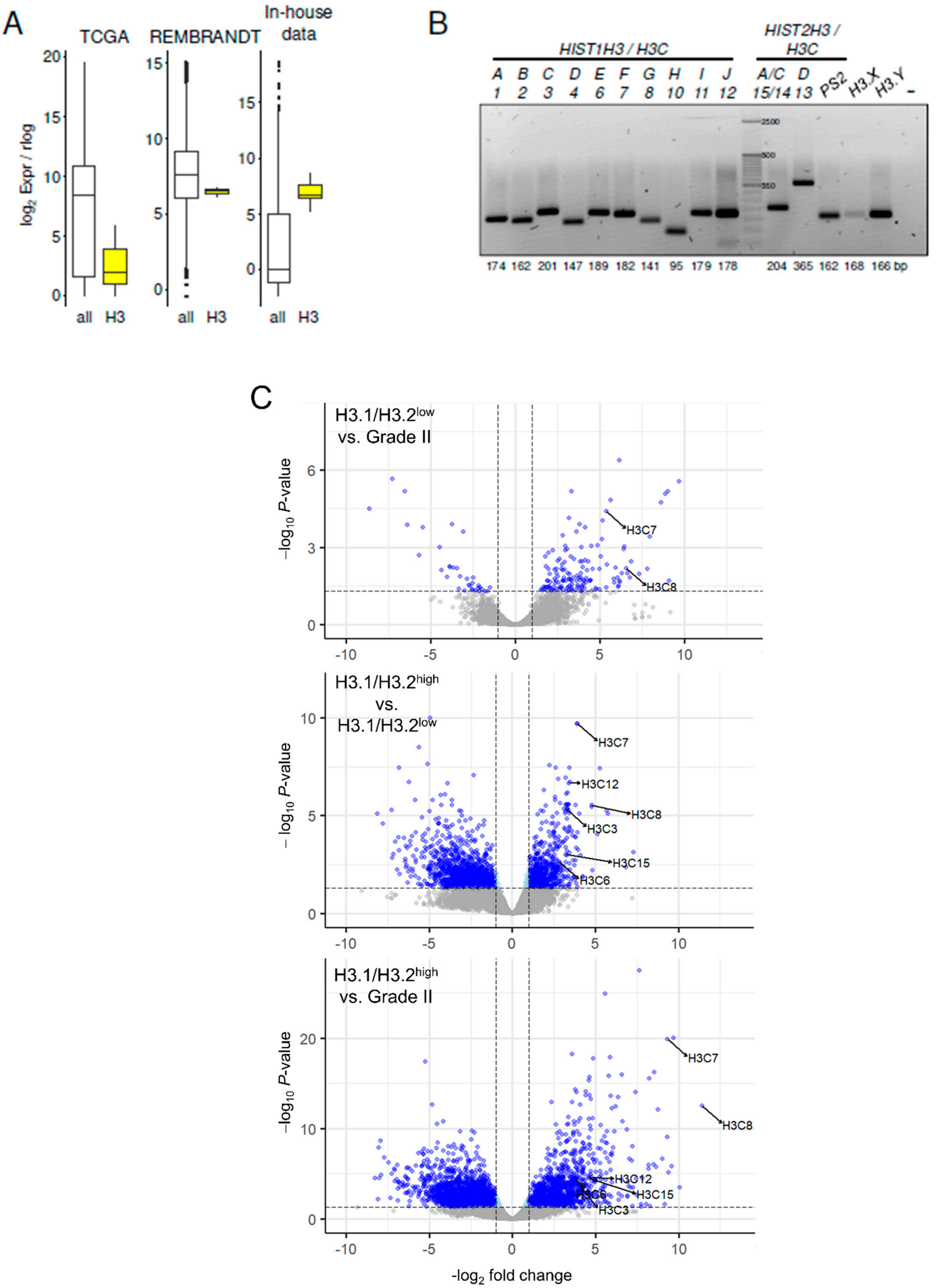

Figure S5. Additional information regarding the expression of canonical histone H3 variants. (**A**) Mean gene expression levels of canonical histone H3 variants in mRNA-seq (TCGA), Affymetrix microarrays (REMBRANDT) and total RNA-seq (own cohort) compared to all genes. The former two approaches obtained very low expression values because of the

polyA selection. (**B**) Gel electrophoresis of PCR products of the intronless canonical histone H3 genes in leukocyte gDNA. Despite being independent genes, *H3C15* / *HIST2H3A* and *H3C14* / *HIST2H3C* share 100% homology in their coding sequence. -, without primers. Numbers indicate the size of the amplicons. (**C**) Volcano plots of the differential expression analysis of the pairs H3.1/H3.2^{high} vs. H3.1/H3.2^{low}, H3.1/H3.2^{high} vs. Grade II and H3.1/H3.2^{low} vs. Grade II. Position of the most relevant histone 3 genes (those significant in the RT-qPCR assays of Figure 4A) are indicated: only *H3C7/HIST1H3F* and *H3C8/HIST1H3G* was significant in the comparison H3.1/H3.2^{low} vs. Grade II, and the rest of genes are not depicted in aims of clarity.

References

1. Esteller M, Hamilton SR, Burger PC, Baylin SB, Herman JG (1999). Inactivation of the DNA repair gene O6-methylguanine-DNA methyltransferase by promoter hypermethylation is a common event in primary human neoplasia. *Cancer Res.* 59: 793-797.
2. Boulay JL, Miserez AR, Zweifel C, Sivasankaran B, Kana V, Ghaffari A *et al* (2007). Loss of NOTCH2 positively predicts survival in subgroups of human glial brain tumors. *PLoS One.* 2**:** e576.
3. Gil-Salu JL, Nieto A, Rodriguez-Gutierrez JF, Almarcha J (2007). [Allelic loss at 1p/19q analysis in brain tumors of glial lineage]. *Neurocirugia (Astur)* 18: 285-293; discussion 293.
4. Reifenberger G, Louis DN (2003). Oligodendroglioma: toward molecular definitions in diagnostic neuro-oncology. *J Neuropathol Exp Neurol* 62: 111-126.
